# Thrombin Generation and Cancer: Contributors and Consequences

**DOI:** 10.3390/cancers11010100

**Published:** 2019-01-16

**Authors:** Caroline J. Reddel, Chuen Wen Tan, Vivien M. Chen

**Affiliations:** 1 ANZAC Research Institute, University of Sydney, Concord 2139, Australia; creddel@anzac.edu.au (C.J.R.); tan.chuen.wen@singhealth.com.sg (C.W.T.); 2 Department of Haematology, Singapore General Hospital, Singapore 169608, Singapore; 3 Department of Haematology, Concord Hospital, Concord 2139, Australia

**Keywords:** cancer, thrombosis, thrombin generation, platelets, procoagulant platelets, extracellular vesicles, neutrophil extracellular traps

## Abstract

The high occurrence of cancer-associated thrombosis is associated with elevated thrombin generation. Tumour cells increase the potential for thrombin generation both directly, through the expression and release of procoagulant factors, and indirectly, through signals that activate other cell types (including platelets, leukocytes and erythrocytes). Furthermore, cancer treatments can worsen these effects. Coagulation factors, including tissue factor, and inhibitors of coagulation are altered and extracellular vesicles (EVs), which can promote and support thrombin generation, are released by tumour and other cells. Some phosphatidylserine-expressing platelet subsets and platelet-derived EVs provide the surface required for the assembly of coagulation factors essential for thrombin generation in vivo. This review will explore the causes of increased thrombin production in cancer, and the availability and utility of tests and biomarkers. Increased thrombin production not only increases blood coagulation, but also promotes tumour growth and metastasis and as a consequence, thrombin and its contributors present opportunities for treatment of cancer-associated thrombosis and cancer itself.

## 1. Introduction

**Figure 1:**
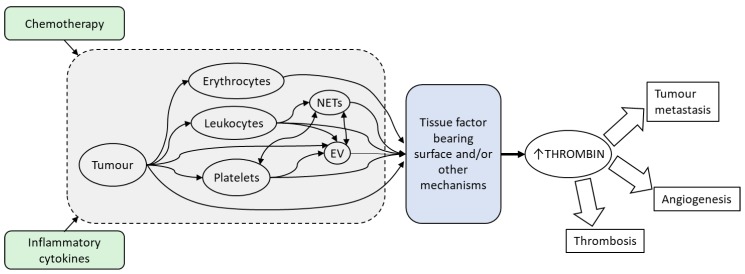
The high occurrence of cancer-associated thrombosis is associated with elevated thrombin generation. Tumour cells increase the potential for thrombin generation both directly, through the expression and release of procoagulant factors, and indirectly, through signals that activate other cell types and components including platelets, leukocytes, erythrocytes, extracellular vesicles (EVs) and neutrophil extracellular traps (NETs). Chemotherapy and the prevailing inflammatory milieu caused by the presence of cancer can stimulate tumour cells and other host cellular components to be procoagulant. Many of these factors potentiate thrombin generation through the expression of tissue factor bearing surfaces that mediates the assembly of coagulation factors essential for the formation of thrombin in vivo. Elevated thrombin production not only increases the risk of thrombosis, but also promotes tumour growth and metastasis and as a consequence, thrombin and its contributors present opportunities for treatment of cancer-associated thrombosis and the underlying cancer.

Patients with cancer are at high risk of pathological thrombosis with risk often exacerbated during cancer treatments. Central to thrombosis is thrombin, the serine protease responsible for the activation of platelets and the conversion of fibrinogen to fibrin. Markers of thrombin generation (both potential for ex vivo thrombin generation [1,2,3,4] and biomarkers indicating previous in vivo thrombin generation [5]) are elevated in patients with cancer, higher in malignant versus benign tumours [6], and negatively predict survival [7]. The implications of elevated levels of thrombin are far-reaching, as this not only indicates a hypercoagulable state and resultant increased risk of cancer-associated thrombosis [5,8] but also promotion of tumour growth and metastasis (reviewed in [9,10,11]). Many factors, both circulating and tumour-direct, contribute to this increased thrombin generation (Figure 1). This review will examine the major contributing factors.

## 2. Thrombin Generation Requires Activation of the Coagulation System and Membrane Surface Interaction

In haemostasis, injury to the endothelium leads to the exposure of factors such as tissue factor (TF) and von Willebrand factor which signals tissue damage and triggers a cascading activation of coagulation factors and recruitment of platelets to the site of injury (reviewed in [12]). The coagulation factors, including prothrombinase complex (FVa and FXa), are assembled on a negatively charged phospholipid surface. Prothrombinase cleaves prothrombin, releasing thrombin and prothrombin fragments 1+2, and eventually resulting in a burst of thrombin which converts fibrinogen to a fibrin clot. Thrombin is inhibited by antithrombin and is released in the form of thrombin-antithrombin (TAT) complexes.

Generation of thrombin thus requires a negatively charged phospholipid surface. During normal haemostasis, the surface is provided by phospholipid externalisation (predominantly phosphatidylserine) on the membrane of activated platelets and/or cell-derived extracellular vesicles (EVs). In cancer, this membrane surface may also be tumour-derived. EVs are small, submicron circulating components in the blood stream consisting of plasma membranes and cytosolic contents derived from the cell of origin. They include microvesicles (<1000 nm membrane-derived EVs, also called microparticles), exosomes (<150 nm vesicles derived from multivesicular bodies), and apoptotic vesicles (<5 μm vesicles released from dying cells) and are released by many cellular sources including platelets, endothelial cells, red blood cells, leukocytes and cancer cells [13,14]. All these factors can be quantified in terms of their expression levels as well as their functional procoagulant activities. Current understanding in the EV field is that the sub-types cannot easily be distinguished from each other [15], and therefore this review applies the term EV to all such vesicles regardless of the nomenclature used in the cited articles.

Excessive thrombin generation and fibrin clot formation contributes to pathological thrombus. There are many factors that, when altered, can lead to the abnormal production of thrombin, many of which are affected by cancer.

## 3. Patients with Elevated Thrombin Generation are at Increased Risk of Cancer-Associated Thrombosis

The potential for thrombin generation in patient plasma can be measured ex vivo by using a thrombin-specific substrate to measure the thrombin burst generated after exposure of plasma to an exogenous stimulant (usually tissue factor) in the presence or absence of blood cells (i.e., whole blood, platelet-rich plasma or cell-free plasma) [16]. The most standardised such test is the Calibrated Automated Thrombogram (CAT, Thrombinoscope BV, Maastricht, The Netherlands), which uses a fluorescent substrate. Technothrombin^®^TGA (Technoclone, Vienna, Austria) is similarly based on a fluorescent substrate. The ThromboPath assay (Instrumentation Laboratory, Bedford, OH, USA) is a similar chromogenic assay but which measures the effect of endogenous activated protein C (APC) in reducing thrombin generation [17].

Only a few prospective studies have assessed the association between elevated thrombin generation and cancer-associated thrombosis. The Vienna Cancer and Thrombosis Study (CATS) measured thrombin generation by the Technothrombin assay in platelet-free plasma in 1033 cancer patients, 77 of whom developed venous thromboembolism (VTE) within 2 years, and found that elevated thrombin generation (peak thrombin in the 75th percentile of the study population) was associated with an increased risk of VTE (hazard ratio of 2.1) [8]. Several smaller studies using the CAT assay have demonstrated similar findings to the CATS study; in 88 patients with malignant gynaecological cancers, thrombin generation was higher in the eight patients who went on to have a VTE after surgery [6], and in 36 multiple myeloma patients followed for 2.5 years, thrombin generation was higher in patients who later had thrombotic events than in those who did not [18]. The potential for thrombin generation [3] and resistance to protein C [19] appears to increase during treatment, corresponding with the increased risk of VTE. However, not all studies show the same association. In a study of 524 breast cancer patients (the CAVECCAS study), in which 56 patients developed a thrombus after venous catheter insertion, thrombin generation parameters by the CAT assay (and indeed any other biomarkers studied) were not predictive of this specific thrombus development [20]. Further prospective studies are needed to determine the predictive value of thrombin generation in different populations with cancer.

While the rapid inhibition of thrombin when generated in vivo means that it is not possible to capture real time thrombin formation in patients, assays are available that show evidence of previous thrombin generation by measuring the by-products of thrombin generation, such as prothrombin fragment 1+2 (F1+2) and thrombin-antithrombin (TAT) complexes. Increased risk of thrombosis is associated not only with increased propensity for thrombin generation but also with elevation of these markers of previous thrombin generation in vivo. In 821 patients from the Vienna Cancer and Thrombosis Study (62 with VTE within 8 months to 2 years) there was an increased risk of VTE associated with elevated F1+2 (HR of 2) and especially with elevated levels of both F1+2 and D-dimer (a product of fibrin degradation) [5]. High D-dimer levels have been identified in a number of studies as predictive of cancer-associated VTE (reviewed in [21]). High pre-operative TAT complexes predicted post-operative deep vein thrombosis (DVT) in 117 patients with solid tumours (nine with DVT) [22]. Taken together, patients at high risk of thrombosis are already generating high levels of thrombin even before potentially fatal events take place.

## 4. Tumour-Specific Factors Contributing to Thrombin Generation

The risk of thrombosis in cancer is associated with factors derived both from the tumour itself and host-related factors, illustrated by studies in which human tumours are introduced into murine models [23,24]. These models allow for isolation of host versus tumour elements by specific blocking of human and/or murine factors (reviewed in [25]). Some of the most important tumour-specific factors are described below.

### 4.1. Tumour Cells and Tissue Factor

In vitro assays demonstrate that certain tumour cells, including solid cancers [26,27,28,29,30] and haematological malignancies [26,28], can directly support thrombin generation. In some tumours, this is related to expression of TF. Clinical epidemiological studies clearly demonstrate that the rate of cancer-associated thrombosis is influenced by cancer type and this is reflected in the clinical risk scores developed to predict thrombosis in ambulatory patients with pancreatic and gastric cancer weighted as highest risk, and lung, gynaecological, lymphoma, bladder or testicular tumours weighted as high risk compared with other cancers (Khorana, Vienna CATS and CONKO scores [31,32,33]). Tumour cells from certain cancers can directly express TF, triggering thrombin generation, but in clinical prediction studies, VTE and tissue factor expression have only been consistently correlated in pancreatic cancer (reviewed in [21]). In vitro, there is a higher level of expression of TF by pancreatic cancer cells (BXPC3) than breast cancer cells (MCF7), corresponding with a higher thrombin generation potential of BXPC3 cells [34]. The thrombin generation activity of both cell types, and also the breast cancer line MDA-MB-231, is sensitive to inhibition by anti-TF antibody [26,27,29]. In one study, solid tumour cell lines (including pancreatic, ovarian, head and neck and lung) had a higher TF-dependent thrombin generation activity than malignant B cell based haematological cell lines (multiple myeloma, plasma cell leukaemia, and histiocytic lymphoma) [28]. However, this does not hold true for all haematological cancers, as several studies indicate thrombin generation activity of leukaemia cell lines including acute promyelocytic leukaemia (NB4) and three monocytic leukaemia cell lines were highly sensitive to anti-TF inhibition [26,35]. Overall, the expression of TF on tumour cells and its effect on thrombin generation depends on the type of cancer.

The specific causative role of tumour-derived TF in thrombosis has been demonstrated by the introduction of human pancreatic cancer cells (expressing human TF) into mice [23]. In this study, anti-human TF reduced in vivo coagulation activation, indicating the tumour-derived TF was driving coagulation rather than the host. However, murine models with murine host TF and human cancer cell TF also indicate that host TF contributes to thrombosis [24].

### 4.2. Tumour Cell-Derived and Tissue Factor Positive Extracellular Vesicles

Circulating cancer cells also release EVs, providing another surface that contributes directly to thrombin generation in cancer. EV-free plasma is not able to generate thrombin [36], and cancer cell-derived EVs directly support thrombin generation, shown by in vitro studies of EVs released from leukaemia cell lines [35,37], prostate cancer cell lines [38] and breast and pancreatic cancer cell lines [39]. Many in vitro studies have linked expression of TF on EVs with their procoagulant potential (as measured by various functional assays such as thrombin generation performed in EV-enriched samples) [35,37,39,40,41]. Circulating TF-positive EVs (TF+EVs) have been seen in leukaemia [37], breast [39], pancreatic [39,42] and lung [42] cancer patients.

In vivo murine studies also support a causative role for cancer cell-derived EVs in thrombus formation. EVs derived from lung cancer cells [42] and breast cancer cells [43] infused into mice promote procoagulant activity in vivo. Cancer-derived TF+EVs can be found incorporated into thrombi of both ferric chloride and laser injury cremaster models of thrombi [44,45]. Furthermore, studies show that thrombus size in murine models is increased with infusion of pancreatic cancer cell-derived EVs or endogenous generated EVs in orthotopic models in a TF-dependent manner, with size and number of occlusive thrombi dependent on TF levels of the EVs [41,45]. EV-enhanced thrombosis is reduced by TF inhibition [41] or by prevention of EV incorporation into thrombus [42]. Furthermore, the thrombotic phenotype induced by TF+EV infusion is suppressed by direct thrombin inhibition with hirudin, indicating that the mechanism is related to thrombin generation [45].

When it comes to clinical samples, the evidence for and against the contribution of TF+EVs to increased thrombin generation in cancer thrombosis is more heterogeneous and mostly does not distinguish between tumour-specific EVs and EVs released by other cell types. Several clinical studies have identified an increased TF expression and/or activity on EVs in cancer patients [46,47,48,49,50,51,52] and especially in those who have previously had a thrombotic event (e.g., [51,53,54,55]). However, fewer studies have correlated TF levels with thrombin generation. Early studies have shown correlation between TF+EVs and activation of coagulation measured by D-dimer levels in colorectal cancer and early stage prostate cancer [52,56]. In contrast, in a small study of pancreatic cancer patients, EV-dependent thrombin generation in whole plasma was TF-dependent in only 1 of 13 cases [57], suggesting non-TF dependent mechanisms are also important in EV-associated thrombin generation in cancer.

Prospective studies in cancer using EV-dependent thrombin generation are extremely limited. In a small group of newly diagnosed acute leukaemia patients, both elevated EV-dependent thrombin generation activity and TF+EV activity (using the Zymuphen “MP-TF” activity assay) was associated with risk of thrombosis in the first week of treatment [58]. Prospective studies more generally investigating TF+EVs and/or TF-dependent procoagulant activity have produced varied results [21]. A recent trial showed an increased risk of VTE (HR 2.0) with elevated TF+EV-dependent procoagulant activity (based on FVII dependence in a fibrin generation test) prospectively measured in 648 cancer patients (40 of whom developed VTE within 6 months), and this risk was most pronounced in pancreatic cancer patients (HR 4.1) [59]. In a group of 43 cancer patients, TF-mediated procoagulant activity (factor Xa generation) on EVs was higher in five patients who developed VTE within six months than in the non-VTE group [60], and in another group of 60 cancer patients without VTE at the time of enrolment, four patients developed thromboembolic disease within a year and all were positive for TF+EVs [55]. In 117 patients with pancreatobiliary cancers, elevated TF activity on EVs (“MP-TF activity assay,” in which large EVs were pelleted and used to trigger FXa generation in the presence or absence of an anti-TF antibody) was associated with VTE (OR 1.4) and lower survival [61]. EV-associated TF activity (using a similar “MP-TF activity assay”) was similarly associated with lower survival in 50 patients with pancreatic or breast cancer [51].

A limitation of much of the current literature around EV contribution to thrombin generation in cancer is that many studies rely on differential centrifugation alone to separate EVs. While these methods enrich EVs they result in impure preparations [62,63]. Another problem remains that of standardisation of TF+EV thrombin generation measurement between laboratories. While commercial assays are available to measure TF+EV coagulant activity, the sensitivity is relatively low [64]. Methodologies are variable with some studies isolating large EVs using ultracentrifugation prior to measuring ability to trigger coagulation, while others measure TF+EV coagulant activity directly in the plasma after recalcification. Assays are not yet standardised enough to allow measurements of tumour-derived TF+EV activity to guide clinical decisions.

## 5. Host-Specific Factors Contributing to Thrombin Generation

Tumour cells also hijack and alter host-specific factors in ways that promote thrombin generation and thrombosis. This section will address some of the relevant changes in host blood cells and host EVs and the inflammatory system.

### 5.1. Platelet Procoagulant Activity and Platelet-Derived EV

#### 5.1.1. Platelets and Platelet-Derived EV in Haemostasis

Platelets play an essential role in thrombosis in both haemostatic and pathological conditions, including cancer-associated thrombosis. There is cumulative evidence demonstrating platelet heterogeneity with the existence of platelet subpopulations after stimulation with strong agonists [65,66,67,68,69]: aggregatory platelets contribute to the formation of platelet aggregation via activation of GPIIb-IIIa whilst procoagulant platelets participate in the coagulation processes leading to thrombin generation. The subset that form procoagulant platelets respond to potent agonist stimulation by an increase in intracellular Ca^2+^ which leads to externalisation of phosphatidylserine (PS) on the outer plasma membrane [65,67,69,70] and supports prothrombinase activity to generate the thrombin burst [65,69] (see Section 2). This localises the fibrin formation to the platelet aggregate and stabilises the thrombus.

Platelet-derived EVs, identified by glycoprotein receptors e.g., CD41 and CD42b, are thought to account for the majority of EVs in circulation [71]. Like activated platelets, they typically express platelet activation markers and PS on their outer membrane, facilitating the assembly of coagulation factors and thereby contributing to thrombin production. Some studies have found TF+EVs to co-express CD41 leading to speculation that tumour EVs may bind to platelets, then be re-shed as TF+platelet EVs [41,52], and suggesting that levels of TF activity in a combined platelet and EV sample may be a more accurate measure of thrombotic risk in patients with cancer compared with the TF activity of tumour EVs alone. Importantly, platelet-derived EVs are produced during the formation of procoagulant platelets, effectively increasing the membrane surface area capable of supporting thrombin generation [72,73,74].

Procoagulant effects of platelets and platelet-derived EVs can be assessed by several methods. As the PS exposure on the external membrane of platelets is a prerequisite for the assembly of coagulation factors on platelets and their associated EVs, flow cytometry-based assays to detect PS expression are often used as a surrogate for measurement of procoagulant platelets. Confocal microscopy can be used to visualise PS exposure as well as the binding of FVa and FXa on the membranes of platelets and EVs [75]. Procoagulant activity contributed by platelets and platelet EVs can be assessed by commercially available functional coagulation tests such as the CAT assay (see Section 3) and STA Procoag-PPL assay (Diagnostica Stago, Asnieres sur Seine, France) (using platelet-free but EV-containing plasma to assess procoagulant activity contributed by EVs). In addition, procoagulant activity of isolated platelets or platelet EVs can also be measured with in-house assays using either a single stage clotting assay or a 2-stage amidolytic assay to quantify the generation of thrombin and FXa [75,76,77]. Recently, a novel assay utilising GSAO [(4-(N-(S-glutathionylacetyl)amino)phenylarsonous acid] and P-selectin permits the direct identification of procoagulant platelets in whole blood by flow cytometry [78] and this may be useful in future studies of cancer thrombosis.

#### 5.1.2. Platelets and Platelet-Derived EV in Cancer-Associated Thrombosis

Cancer causes increased platelet activation and aggregation, and these effects have been extensively reviewed [79,80,81,82,83]. Certain malignant cells can promote platelet activation through multiple pathways including direct activation by TF on the tumour or tumour EVs, tumour-derived thrombin generation leading to platelet activation via cleavage of platelet thrombin receptors PAR1 and PAR4, and tumour-derived cytokine and metalloprotease secretion (reviewed in [80,81]). In certain cancers, platelet activation and thrombosis rates are related to aberrant expression of platelet activating ligands on tumour cells such as the CLEC-2 ligand, podoplanin, on both glioblastoma and acute promyelocytic leukaemia [84,85]. Once activated, there is a large potential PS surface contribution from platelets for thrombin generation, as described above.

Procoagulant platelets and platelet-derived EVs and their associated enhanced procoagulant activities are increased in several solid organ malignancies. Phosphatidylserine-positive (PS+) procoagulant platelets are elevated in patients with gastric cancer [86], non-small cell lung cancer [87] and colon cancer [76]. Platelet-derived EVs are also elevated in patients with various cancers including gastrointestinal, breast, lung and prostatic malignancies [88], non-small cell lung cancer [87], colon cancer [76] and cutaneous malignant melanoma [89]. Both procoagulant platelets and platelet-derived EVs associate with tumour state. For example, both increase with stage of colon cancer [76], procoagulant platelets decrease with resection of gastric tumour [86] and platelet-derived EVs are higher in malignant breast [90] and ovarian [91] cancer compared with benign tumours. Platelet EV numbers associate with prior VTE in patients with cancer; patients with VTE had higher TF-expressing EVs than those without, and TF-expressing EVs were strongly correlated with platelet-derived EVs [88]. In another group of patients with soft tissue sarcoma in which the overall population of EVs was increased compared to healthy controls, activated platelet-derived EVs (defined as CD62P and CD63 positive population) were significantly elevated in patients with history of VTE than those without [82].

By performing functional procoagulant activity assays, several of these studies have additionally shown a link between elevated levels of procoagulant platelets or platelet-derived EVs and elevated thrombin generation. For example, both platelets and platelet-derived EVs from patients with colon cancer had elevated factor Xa and prothrombinase formation and thus thrombin generation [76], and platelets from patients with gastric cancer had enhanced procoagulant functions with higher prothrombinase activity [86]. Levels of platelet-derived EVs in cutaneous malignant melanoma correlated with procoagulant potential as measured by STA Procoag-PPL assay [89]. However, this relationship cannot be extrapolated to all cancers as plasma samples containing elevated platelet-derived EVs from patients with breast cancers did not show a correlation between levels of EVs and thrombin generation [90].

Perhaps the most consistent evidence for contribution of platelet-derived thrombin generation to thrombotic risk is seen in essential thrombocythaemia, a Philadelphia-negative myeloproliferative neoplasm (MPN) characterised by clonal proliferation of the megakaryocytic lineage within the bone marrow and elevated platelet count in peripheral blood. Up to 20% of patients have a history of thrombosis at diagnosis, and the ongoing rate of thrombus is between 1% and 4% per year despite current standard of care therapy [92,93]. Elevated levels of PS+ procoagulant platelets have been shown in several studies of patients with essential thrombocythaemia [94,95], and the related disorders of polycythaemia vera and myelofibrosis [96,97]. Furthermore, in these diseases, using identical total number of platelets as controls, patients’ platelets (containing higher PS+ platelet subset) support higher thrombin and fibrin generation [75] suggesting a mechanistic link between the observed hypercoagulability and this platelet subset. Patients with both essential thrombocythaemia and polycythaemia rubra vera have heightened thrombin generation, measured in platelet-rich-plasma and platelet isolates, compared to controls. These findings support a causative role of procoagulant platelets in elevated thrombin generation contributing to hypercoagulability in MPN [98]. Patients with MPN and prior history of thrombosis also exhibit raised EV levels compared with patients without thrombosis [96], however the contribution of EVs to thrombin generation in MPN is less clear. Unlike the findings in platelet-rich plasma, two studies have found no increase in thrombin generation using the CAT assay in platelet-free plasma [98,99], indicating that EVs may have a less significant functional role in thrombin generation in MPN. Interestingly, the same group found that platelet-free plasma from essential thrombocythaemia patients had increased peak thrombin (a CAT assay parameter) when thrombin generation was assayed in the presence of activated protein C indicating that the EV contribution to thrombin generation may be acting via the protein C pathway.

Overall, these findings point to a complex relationship between procoagulant platelets and platelet-derived EVs as potential markers of and contributors to cancer and cancer-associated thrombosis.

### 5.2. Leukocyte-Derived Extracellular Vesicles and Tissue Factor

Elevated systemic levels of leukocytes are associated with cancer, and with the risk of VTE (reviewed in [100]), and several studies have identified elevated levels of leukocyte-derived EVs in cancer [101,102,103]. However, little is currently known about the role of leukocyte-derived EVs, especially regarding their contribution to thrombosis. In healthy subjects and other diseases such as atherosclerosis, leukocyte-derived EVs play a wide variety of roles, not limited to procoagulant activities (reviewed in [104]).

Monocytes do not usually express TF, but highly express it in response to stimuli [105]. Capacity of monocytes to express active TF is increased in a range of thrombotic diseases (reviewed in [106]), including cancer. Elevated TF activity was observed in response to lipopolysaccharide stimulation in monocytes from bladder, prostate, breast and colorectal cancer patients compared with cells from healthy controls [107]. Monocytes from a group of patients with essential thrombocythaemia had higher levels of circulating TF than healthy controls, and within this patient group, those with previous VTE had increased capacity to express TF in response to lipopolysaccharide [108]. Further, monocytes from patients with polycythaemia vera had an increased capacity to express TF in response to endotoxin stimulation, which correlated with circulating F1+2 [109]. There is some evidence for a causative link between monocyte TF and thrombosis. In a rabbit model, leukocytes extracted from animals treated by injection of endotoxin which expressed a high level of TF activity were injected into healthy animals, causing widespread clots (compared with low TF leukocytes, which did not cause clots) [110].

In vitro studies demonstrate that stimulated monocytes release TF+EVs [111,112,113] and that monocyte-derived EVs have the capacity to trigger thrombin generation [112,113]. The thrombin generation activity of monocyte-derived EVs, like tumour-derived EVs, appears to be TF-dependent [113]. Overall, monocyte-derived EVs, in addition to tumour-derived EVs, could therefore contribute to the increased risk of thrombosis in cancer patients with high levels of EV-associated TF activity.

### 5.3. Erythrocytes

There is evidence to suggest that some erythrocytes with PS exposure could enhance thrombin generation in the setting of malignancy. There are more PS+ erythrocytes and erythrocyte-derived EVs from patients with polycythaemia rubra vera than healthy controls and those with secondary polycythaemia, and these PS+ erythrocytes and EVs exhibit increased procoagulant activity [114]. In addition, some chemotherapies could potentiate their procoagulant activity through erythrocytes: Zhou et al. showed that daunorubicin could increase PS exposure of erythrocytes in patients with acute myeloid leukaemia and these erythrocytes in turn exhibit enhanced thrombin generating potential [115].

### 5.4. Inflammation

Inflammation is central to the development and progression of cancer (reviewed in [116,117]). The tumour microenvironment contains neutrophils, dendritic cells and tumour-associated macrophages, and tumour cells release cytokines and chemokines. Leukocytes form part of the thrombotic response (reviewed in [118]) and there is a close interplay between inflammation and coagulation (reviewed in [119]). Some of the factors contributing to this are discussed below.

#### 5.4.1. Neutrophil Extracellular Traps (NETs)

Activated neutrophils can expel neutrophil extracellular traps (NETs), networks of DNA, histones and cellular proteins which play a role in host defence, but also have detrimental effects in various diseases, including an association between markers of NETs and thrombosis/elevated thrombin generation/risk of VTE (e.g., sepsis [120,121], inflammatory bowel disease [122] and Cushings disease [123]). NETs are found in thrombotic material from patients [124,125], and in mice, blocking NET formation by knocking out protein arginine deiminase 4 (PAD4) greatly reduces thrombosis caused by an inferior vena cava ligation model of DVT [126] and reduces intravascular thrombin activity in a model of sepsis [127]. Ex vivo experiments using neutrophils from both mice [128] and humans [120,129] show that NETs can trigger thrombin generation which is abolished by DNase treatment and factor XII inhibition [120,128]. One study found that cell-free DNA alone triggered thrombin generation, while intact NETs did not [130]. Histones infused into healthy mice increased markers of thrombin generation in vivo (and not when infused together with an antibody against histones) [131] and may have direct effects on thrombin generation or act indirectly e.g., via expression of TF on other cell types (reviewed in [132]). NETs may also express TF themselves (e.g., in the atherosclerotic plaque from ST-elevation myocardial infarction patients [124] or in an in vitro model of antineutrophil cytoplasmic antibody–associated vasculitis [133]) which could contribute to their procoagulant activities.

Circulating markers of NETs are increased in patients with cancer [125,134] and neutrophils from patients with cancer have a higher capacity to release NETs in vitro (e.g., gastric cancer [135], colorectal cancer [136], stage I/II oral squamous cell carcinoma [137], chronic lymphocytic leukaemia [138], but not Philadelphia-negative MPNs [139]). Mouse models of chronic myelogenous leukaemia, mammary and lung carcinoma also have increased sensitivity for NET formation [140]. Pancreatic cancer cells can induce NET formation from polymorphonuclear neutrophils in vitro [141] and in a mouse model of pancreatic cancer, blocking NET formation reduced hypercoagulability (as measured by thromboelastogram) and circulating TF [142]. In 936 patients from the Vienna Cancer and Thrombosis Study, citrullinated histone levels were significantly higher in the 89 who developed VTE within 2 years, with a SHR of 1.13 per 100 ng/mL increase. Cell-free DNA in the highest quartile was also associated with an increased risk of VTE [143].

NETs interact with both platelets (reviewed in [144]) and EVs. They can stimulate platelet activation [142] and procoagulant platelet formation [145], are involved in platelet adhesion to neutrophils [128], and are stimulated by P-selectin [146], which is highly expressed by activated platelets and released onto platelet-derived EVs. NETs may also act synergistically with EVs to promote procoagulant activity [128]. Furthermore, tumour EVs have been shown to localise to NETs with reduction in thrombus size when NET/EV interactions are disrupted [45]. Strategies to reduce NETs may be an additional mechanism to reduce thrombotic risk in cancer.

#### 5.4.2. Pro-Inflammatory Mediators

Thrombin activates the complement system (reviewed in [147]), and cytokines and complements can activate the coagulation system. For example, IL-1β and TNF-α cause an increase in TF expression by vascular cells [148,149]. Identifying a clinical link between cytokines and thrombosis risk is not clear. The Vienna Cancer and Thrombosis Study found no association between VTE and any soluble inflammatory marker (except for a trend for the association of IL-1β and IL-6 with VTE in pancreatic cancer) [150], although in another study, IL-6 was found to be an independent predictor of VTE in 200 patients with ovarian cancer (HR 8.9) [151]. In ovarian cancer, IL-6 increases thrombopoietin expression by hepatocytes, which increases platelet production by megakaryocytes (reviewed in [25]) providing an additional link between inflammation and platelet-dependent thrombin generation. Elevated E-selectin (an adhesion molecule on endothelial cells activated by cytokines) was an independent risk factor (OR 1.41) for postoperative DVT in a mixed group of cancer patients [152]. Infusion of IL-6 into patients with metastatic renal cell cancer caused elevated markers of thrombin generation [153], and in a mouse model of cancer cachexia with high, TF-independent thrombin generation, blocking tumour-derived IL-6 led to a decrease in thrombin generation by the CAT assay [154], implying a causative association between elevated cytokines and thrombosis in cancer.

## 6. Chemotherapy Effects on Thrombin Generation

Chemotherapy used in the management of cancer is considered a major factor for hypercoagulability in cancer patients. The direct tumour effect on thrombin generation may be exaggerated after chemotherapy, providing one explanation for the ensuing heightened procoagulant state. For example, cisplatin, carboplatin, gemcitabine and paclitaxel all increased TF activity on lung cancer cells in vitro [155] and cisplatin treatment may cause an increase in TF in germ cell tumour cells [156]. It is suspected that one mechanism for increased risk of VTE during chemotherapy is apoptotic vesicles released upon death of the tumour cells, which appear to be more procoagulant than microvesicles and are sensitive to anti-TF inhibition (recently reviewed in [157]). Cisplatin and gemcitabine also increased TF antigen and activity on monocytes in vitro [155]. The increased VTE risk of gemcitabine and platinum based therapies are incorporated into the PROTECHT clinical prediction risk score [158].

Platelets are also implicated as critical contributors to this thrombogenic process after chemotherapy. Doxorubicin-treated platelets displayed higher levels of phosphotidylserine, increased intracellular calcium and generated more thrombin than untreated controls [159], and doxorubicin increased platelet mitochondrial inner transmembrane potential depolarisation in a concentration and time dependent manner [160], consistent with the phenotype of procoagulant platelet. These procoagulant platelet effects also follow doxorubicin exposure in an in vivo rat thrombosis model [159]. Patients with non-small cell lung cancer had increased procoagulant platelets and platelet-derived EV levels after treatment with combination gemcitabine and cisplatin chemotherapy [87]. Moreover, there was enhanced platelet-dependent thrombin and fibrin formation observed post-chemotherapy and use of aspirin partially attenuated this enhanced platelet-induced procoagulant activity in vitro [87]. Further, arsenic therapy in the treatment of acute promyelocytic leukaemia has been shown to potentiate PS exposure on platelets. These platelets displayed increased intracellular calcium and generated more thrombin and were related to increased thrombosis in a rat model of thrombosis [161]. The fact that these effects are mediated by procoagulant platelets suggests a potential novel intervention to decrease hypercoagulability after chemotherapy.

## 7. Thrombin Role in Tumour Biology/Metastasis

The role thrombin plays in the protection and promotion of tumours has been comprehensively reviewed (e.g., [9,10,11]). There is considerable evidence from animal models that thrombin augments tumour growth and metastasis (reviewed in [162]). For example, thrombin increases metastases when tumour cells are injected into mice in its presence [163], reduction of circulating prothrombin levels reduces tumour growth in a colon cancer model [164] and inhibition of thrombin in a breast cancer model reduces bone metastasis [165]. The thrombin receptors protease activating receptor (PAR)-1 and -2 are expressed on a broad range of tumour cells, and activation of these receptors has effects which include tumour growth, cell proliferation, migration, invasion and increased inflammation and angiogenesis (reviewed in [166]). Further, thrombin appears to alter gene expression of tumour cells to promote oncogenesis (reviewed in [167]). Tumour cells may also express TF on the cell surface to aid the process of epithelial-mesenchymal transition and thus increasing their metastatic potential [168].

In addition to these direct functions, thrombin promotes cancer progression and metastasis via platelets. Thrombin activates platelets [169] and promotes their interactions with tumour cells [163]. The fact that platelets play an important role in tumour growth and metastasis has been thoroughly and recently reviewed [170,171,172,173,174,175,176,177]. Platelet count correlates with poor prognosis (reviewed in [171]) and inhibition of platelets inhibits metastasis in vitro and in animal models (reviewed in [173]). Platelets facilitate tumourigenesis by mechanisms including the secretion of factors (reviewed in [170]) promoting tumour cell proliferation (e.g., transforming growth factor-β1), pro-angiogenic factors (e.g., vascular endothelial growth factor and platelet-derived growth factor) and changes in cellular expression including promotion of the epithelial–mesenchymal transition [171,175]. Platelets also directly interact with endothelial cells to induce angiogenesis [174]. Circulating tumour cells are stabilised by platelet and fibrin aggregates forming around them, protecting them from shear forces and host immunity, and promoting invasion [167,170,171,176]. The promotion of thrombin in cancer thus forms a positive feedback loop, with not only potentially fatal effects on coagulation but also further propagation of the disease.

## 8. Conclusions

There are a number of systemic and tumour-driven changes in cancer which support thrombin generation. Studies in the pathogenesis of cancer indicate a key role for thrombin in tumour progression, however no single assay has been able to predict risk of thrombosis and outcome in all cancers, pointing to the complexity and multi-factorial nature of this phenomenon. Haemostatic tests, such as the thrombin generation assay, show some promise, but these are not yet standardised and to date, there have been few prospective studies that show the association between elevated global thrombin generation and cancer-associated thrombosis.

While evidence is building strongly for a role for tumour- and host-derived EVs in cancer-associated thrombin generation and thrombosis, and as methods become more established and standardised, prospective studies are still needed to determine the association between circulating cells, cell-derived factors and thrombosis. The increased thrombotic risk is also directly contributed to by tumour cells and tumour-educated blood cells, including platelets, indicating the importance of assessing the cellular and EV procoagulant potential. This is a promising field in which understanding the pathophysiology of the drivers of cancer associated thrombin generation may eventually lead to risk scores to predict cancer related thrombosis and targeted interventions that address the individual risk factors driving thrombosis in cancer patients.
